# Data Reconstruction Methods in Multi-Feature Fusion CNN Model for Enhanced Human Activity Recognition †

**DOI:** 10.3390/s25041184

**Published:** 2025-02-14

**Authors:** Jae Eun Ko, SeungHui Kim, Jae Ho Sul, Sung Min Kim

**Affiliations:** Department of Regulatory Science for Medical Device, Dongguk University, Seoul 04620, Republic of Korea; 2018111721@dgu.ac.kr (J.E.K.); 2023126928@dgu.ac.kr (S.K.); 2018111719@dongguk.edu (J.H.S.)

**Keywords:** human activity recognition, HAR, accelerometer, data reconstruction, CNN, spectrogram, recurrence plot, multi-channel plot

## Abstract

Background: Human activity recognition (HAR) plays a pivotal role in digital healthcare, enabling applications such as exercise monitoring and elderly care. However, traditional HAR methods relying on accelerometer data often require complex preprocessing steps, including noise reduction and manual feature extraction. Deep learning-based human activity recognition (HAR) using one-dimensional accelerometer data often suffers from noise and limited feature extraction. Transforming time-series signals into two-dimensional representations has shown potential for enhancing feature extraction and reducing noise. However, existing methods relying on single-feature inputs or extensive preprocessing face limitations in robustness and accuracy. Methods: This study proposes a multi-input, two-dimensional CNN architecture using three distinct data reconstruction methods. By fusing features from reconstructed images, the model enhances feature extraction capabilities. This method was validated on a custom HAR dataset without requiring complex preprocessing steps. Results: The proposed method outperformed models using single-reconstruction methods or raw one-dimensional data. Compared to a one-dimensional baseline, it achieved 16.64%, 13.53%, and 16.3% improvements in accuracy, precision, and recall, respectively. We tested across various levels of noise, and the proposed model consistently demonstrated greater robustness than the time-series-based approach. Fusing features from three inputs effectively captured latent patterns and variations in accelerometer data. Conclusions: This study demonstrates that HAR can be effectively improved using a multi-input CNN approach with reconstructed data. This method offers a practical and efficient solution, streamlining feature extraction and enhancing performance, making it suitable for real-world applications.

## 1. Introduction

As the demand for digital healthcare grows, human activity recognition (HAR) has become imperative for practical applications, such as exercise tracking, fall detection, and elderly monitoring. The activity monitoring plays a crucial role in providing real-time feedback on the behaviors of elderly individuals and those requiring special assistance, supporting both medical rehabilitation and caregiver efforts [1]. Furthermore, applications such as fall detection and posture analysis can swiftly identify actual fall events, helping to prevent falls and reduce related healthcare costs [2].

Numerous HAR systems have been developed using various approaches such as wearable sensors, video cameras, and Kinect sensors. Activity data collected through various sensors are used to recognize both simple and complex activities such as walking, running, sitting, lying, falling, and other daily living activities. In recent years, wearable devices equipped with sensors have gained popularity for monitoring human activities, driven by rapid advancements in sensor [3]. The most common approach for systems utilizing wearable devices is based on data acquired from a tri-axial accelerometer [4]. A tri-axial accelerometer measures increasing speed, tilt, inclination, rotation, vibration, and crash along the x, y, and z axes [5]. These measurements provide valuable data for HAR, where the key lies in feature extraction to identify meaningful patterns and representations related to various movements [6].

Traditionally, HAR methodologies have followed two main steps: feature extraction and pattern classification [7]. The feature extraction process encompasses the selection of features that best represent the characteristics of the accelerometer signals. These features may include measurement such as mean, standard deviations values of the samples in a frame, energy measures, interquartile range, and signal entropy, among others [8]. However, this process is intricate, as it necessitates the manual selection of hand-crafted features to be employed in classification. Consequently, the efficacy of HAR can significantly vary depending on the chosen features. Moreover, since the features are typically determined by human expertise, users are required to possess specialized knowledge and experience in activity recognition [3,9].

Recently, the integration of deep learning into HAR has been conducted, thereby enabling reducing the dependence on the complex manual selection of representative features. R. Mutegeki et al. [10] utilized data from IMU (Inertial Measurement Unit) sensors, including accelerometers, gyroscopes, and magnetometers, which were applied to a CNN-LSTM model. However, the results were derived from relatively simple datasets consisting of only four or six classes. C.A. Ronao et al. [11] applied one-dimensional data obtained from accelerometers and gyroscopes to a CNN through feature engineering. A. Ignatov et al. [12] and F. Nazar et al. [13] combined one-dimensional accelerometer data with statistical features and applied them to a CNN model.

Research on HAR using one-dimensional accelerometer data have exhibited promising results. However, while CNNs perform well on specific types of data, their limitations with one-dimensional data necessitate exploring alternative methods to improve performance [14]. To address these limitations, this study aims to overcome the challenges of deep learning-based HAR using one-dimensional accelerometer data by leveraging two-dimensional representations. Particularly, converting one-dimensional time-series physiological signals into images and applying them to CNN models has proven effective in mitigating measurement noise [15]. Furthermore, the graphical characteristics of two-dimensional representations can aid in extracting prominent features that may not be apparent in raw time-series data by revealing hidden patterns and structural changes [16,17].

In this context, there have been previous studies that convert one-dimensional time-series accelerometer data into two-dimensional image data for use in HAR research. Various studies have demonstrated improvements in HAR performance by transforming time-series data into images using techniques such as Fourier transform [18,19], recurrence plot [7,20,21,22], and multi-channel plot [23], followed by their application in CNN models. Building on these advancements, several research methods such as hybrid, multi-branch, and ensemble HAR were proposed to improve the accuracy of HAR. Several studies have explored sensor fusion using accelerometer, gyroscope, and magnetometer data [5,24,25,26], as well as ensemble methods of two models combined [24,27,28,29,30]. Additionally, research combining time-series data with short-time Fourier transform (STFT) [23,31] has been proposed.

However, these previous studies typically focus on extracting features from a single image and may not fully capture the implicit characteristics that can be derived from various reconstruction methods. Furthermore, pre-processing steps such as normalization and filtering are often conducted before image transformation. This approach does not fully leverage the noise suppression benefits that images can provide. Moreover, the incorporation of additional sensor data, such gyroscope information, often requires pre-processing, which can complicate the process. These studies also generally evaluate their methods on datasets with fewer and simpler classes, which may not reflect the complexity of real-world scenarios.

Inspired by this view, this study reconstructs multiple images from a single accelerometer dataset, allowing for the extraction of a broader range of latent features. These reconstructed images are then utilized as inputs in a multi-input feature fusion CNN for HAR. By directly converting the data into images, we are bypassing the need for traditional signal processing steps such as noise reduction and filtering, simplifying the process while taking full advantage of the noise suppression capabilities inherent in image data. Additionally, moving beyond small, simple datasets, we have collected a large-scale dataset that includes 15 distinct activities, enabling a more thorough performance evaluation in real-world contexts. This study builds upon our previous work presented at AIS-I3S 2024 [31]. The main contributions of this study are as follows:

1. We propose a multi-features fusion CNN architecture with three data reconstruction methods to effectively capture the latent features of time-series data and enhancing feature extraction and classification performance.
2. This study presents a large-scale HAR dataset with 210 subjects and 15 activities, including daily living and fall-related movements, to support real-life action monitoring.
3. We validated the proposed method on our own dataset, demonstrating the potential of multi-input fusion CNN in improving HAR performance.

## 2. Related Works

### HAR with Multi-Feature Fusion Models

Recent advancements in HAR have explored multi-feature fusion techniques to improve classification accuracy, particularly by integrating multiple sensor modalities or different feature representations. Multi-feature fusion approaches leverage additional sensor data or complementary transformations to enhance feature diversity and robustness.

Several studies have proposed combining multiple sensor modalities to improve HAR performance. Ravi et al. [32] transformed accelerometer and gyroscope data into spectrograms and extracted shallow statistical features such as mean, standard deviation, amplitude, skewness, and kurtosis. These spectrogram representations were then fused with the statistical features for classification. Kang et al. [33] assembled skeleton coordinates and time-series data feature vectors using CNNs and the convolutional block attention module. Webber et al. [24] fused accelerometer and gyroscope data at the sensor level, feature level, and decision level. Dogan et al. [5] combined the STFT representations of accelerometer, gyroscope, and magnetometer data. Chai et al. [34] attached IMU and sEMG sensors to the upper and lower body, then applied SVM, KNN, Decision Tree, Random Forest, and CNN-LSTM model ensembles. However, implementing these methods in real-world scenarios poses challenges due to the necessity of multiple physical sensors, which increases system complexity. Additionally, complex preprocessing is required to synchronize and align different sensor data streams, leading to increased computational resources and processing time.

Some studies focused on extracting different feature representations from the same dataset using various models. Sahoo et al. [26] transformed accelerometer data into spectrograms and fused features from MobileNet and EfficientNet. Dua et al. [27] used the same input but applied CNN-GRU with multiple filter sizes for feature fusion. Jain et al. [28] applied one-dimensional CNN and LSTM fusion on raw time-series data. Nedorubova et al. [29] and Almanifi et al. [30] converted time-series data into Continuous Wavelet Transform (CWT) and applied CNN model ensemble for classification. These studies employed a strategy of generating different representation vectors from a single dataset using multiple models and subsequently fusing them. However, as some models may learn similar features, redundant information from the same data could reduce overall efficiency.

Several studies have explored feature fusion between handcrafted features and accelerometer data to enhance HAR performance. N. Bento et al. [35] combined handcrafted features with time-series data using ResNet for feature concatenation and classification. Zheng et al. [23] compared an accelerometer spectrogram with shallow features and spectrogram-only approaches, concluding that the spectrogram-only model achieved better performance. This suggests that the choice of raw data representation significantly impacts model performance, and the handcrafted features may have drawbacks such as subjectivity and a lack of reproducibility, which can affect model generalizability.

Another approach explored by G. Sharma et al. [36] involved contrastive learning by utilizing multi-view attention on spectrograms and time-series accelerometer data. However, since this study relies solely on the original time-series signal and its spectrogram transformation, it may overlook latent features that could be discovered through alternative image transformation methods.

Most previous studies have focused on converting sensor data into a single-image representation and then applying different models for ensemble learning or integrating multiple sensor modalities. In contrast, we take a different approach by deriving multiple representations from a single accelerometer dataset. By extracting latent features from the same data source, we achieve performance improvement without relying on additional sensors or complex multi-model ensembles. Furthermore, our model is designed with a lightweight architecture, utilizing significantly fewer parameters. This not only enhances computational efficiency but also makes real-world deployment more feasible by reducing resource consumption and inference time.

## 3. Materials and Methods

### 3.1. Data Collection

The experiment is performed on our own dataset. Our dataset is acquired by a holter electrocardiography sensor (HiCardi, Mezoo Co., Ltd., Wonju, Republic of Korea) at a 250 Hz sampling rate. The holter electrocardiography patch sensor is attached on the left chest. The Institutional Review Board (IRB) of Yonsei University Health System, Severance Hospital College in South Korea approved the study protocol (No. 1-2023-0006). Informed consent was obtained for all participants enrolled in the data collection. Subjects who voluntarily provided written informed constant prior to participating in the clinical trial, and who are between 20 and 40 years old, without any physical disabilities, and do not experience discomfort in replicating daily activities, exercise postures, or fall scenarios, were included in this study. Exclusion criteria were physical impairments, fear or discomfort in simulating fall postures during fall-related research, and difficulty in wearing the holter electrocardiography sensor. While ECG and accelerometer data were obtained, only accelerometer data were utilized in this study. In total, 210 subjects participated in data collection. The demographic characteristics of the subjects can be found in Table 1, with 115 males and 95 females included in the study.

**Figure 1:**
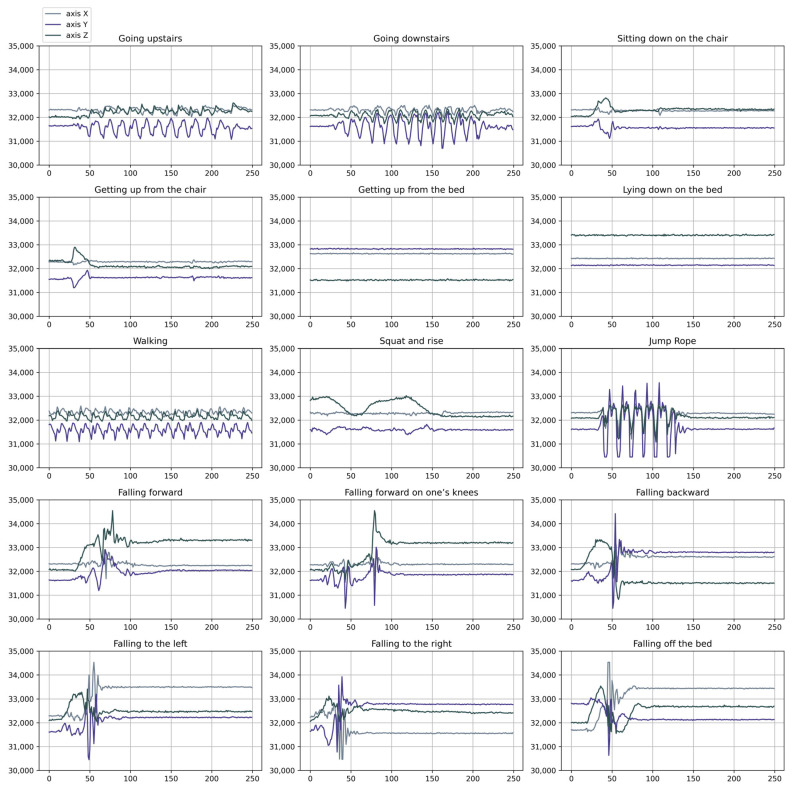
Plots of the 15 activities for a single subject.

This dataset is compiled with two types of movements: 6 classes of falls and 9 classes of daily activities. A total of 15 kinds of daily activities were performed, repeated 5 times. The 15 types of activities are detailed in Table 2 and visualized in Figure 1.

Compared to publicly available HAR datasets [37,38,39,40,41], our dataset offers two key advantages. First, it includes data from 210 subjects, which is significantly larger than most existing datasets, where the number of participants typically ranges from 10 to 30. This larger sample size enhances the generalizability of the models, allowing for a more robust performance across diverse individuals. Second, while many HAR datasets primarily focus on basic daily activities, our dataset comprises 15 activities that were carefully selected to reflect real-life scenarios, including both daily movements and fall-related actions. The incorporation of fall-related activities is particularly relevant for action monitoring and safety-critical applications, where the accurate recognition of such movements is essential.

### 3.2. Data Preprocessing

In this study, we focus on converting one-dimensional time-series data into images, a method proven to effectively mitigate measurement noise [14,15]. By leveraging this conversion, we eliminate the need for additional normalization or filtering processes, which are commonly used in data preprocessing. Instead, we directly segment the raw data for further analysis. In HAR, segments typically range from 4 to 10 s [42]. Using segments instead of single data points is motivated by the fact that the raw inertial measurements fluctuate significantly, making the classification of a single data point impractical [32]. In this study, the data were segmented into 10 s intervals based on the class to which they belonged.

A total of 15,750 samples were collected for this study. Of these, 12,600 samples were used to form the training dataset, and 3150 samples were reserved for the test dataset. Additionally, 3150 samples from the training dataset were further separated for use as a validation dataset. In line with standard practices for HAR performance evaluation, subject-independent evaluation was performed by splitting the data at the subject level. Specifically, the training and test datasets were separated to ensure that the same subjects did not appear in both sets. This approach is critical to avoid the model learning subject-specific motion patterns rather than general activity features. Moreover, it better reflects real-world scenarios where the model must classify unseen data during deployment, capturing the challenges of activity recognition in new, unobserved subjects.

### 3.3. Data Reconstruction

**Figure 2:**
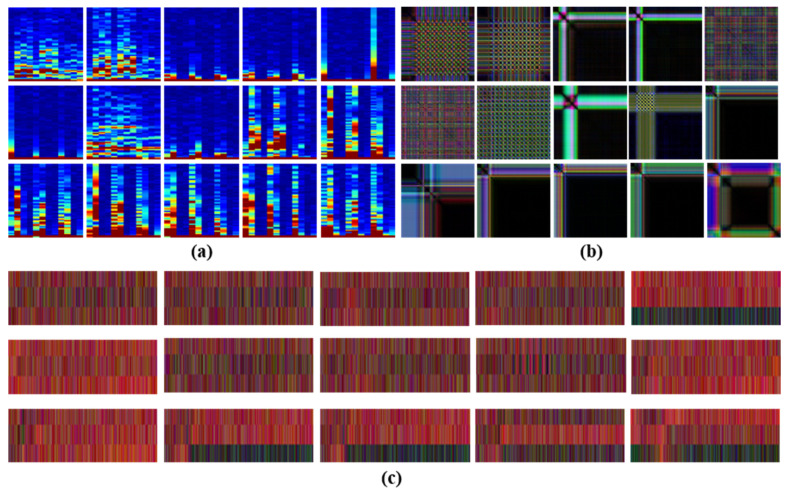
Images reconstructed by (**a**) spectrogram, (**b**) modified RP, and (**c**) modified MP.

The input data for the proposed two-dimensional CNN are required to be of image type. Therefore, accelerometer signals are transformed into images using three different reconstruction methods: spectrogram, modified recurrence plot (RP), and modified multi-channel plot (MP). Figure 2 shows the reconstructed images. To reduce training time and computational cost, each method encompasses information from the x, y, and z axes for the comprehensive input image. Each reconstruction method generates one image, resulting in three input images for each time-series dataset.

#### 3.3.1. Spectrogram

Spectrograms are generated using short-time Fourier transformation (*STFT*). A spectrogram represents an inertial signal *x* as a function of frequency and time. Specifically, it is computed as the magnitude squared of the *STFT*. The *STFT* calculates the Fourier transform separately for each short segment of the signal. This process is expressed as follows:

(1) $$STFT\{x[n]\}\left(m,\omega \right)=\sum_{n}x[n]\omega [n-m]\;exp(-j\omega n)$$

where *x*[*n*] is the signal and ω[*n*] is the window function; *exp*(*−j*ω*n*) is the exponential function, where *j* is the imaginary unit, and ω is the frequency index. The spectrogram is obtained by squaring the magnitude of the STFT:

(2) $$Spectrogram\{x[n]\}(m,\omega )={|X(m,\omega )|\;}^{2}$$

The raw accelerometer signal represents the motion acceleration occurring in the time domain. The spectrogram enables the analysis of the differences among the nearest accelerometer data points in the frequency domain, allowing for the examination of the temporal variation in frequency induced by human movement [42]. In prior studies, window lengths of approximately 1 s [18,23], 5 s [43], 10 s [44,45], and 12 s [46] were used to segment raw inertial time-series data. Considering that the cadence of normal walking is approximately 90 steps per minute (1.5 steps per second) [47], we started our experiments with a 1 s window size and a 50% overlap. For our experiments, spectrograms were generated using window lengths of 1/2/5/10 s, as segments longer than 10 s could not be tested due to the segment length limitation of our dataset. To the best of our knowledge, there has been no prior research that specifically analyzes the optimal window size for effectively converting accelerometer data into spectrograms. Therefore, we conducted an analysis to determine the optimal window size for segmentation. Based on our findings, we used spectrogram images generated with a window size of 10 that produced the best results. Previous studies [32,42,44,48] converted x, y, and z axes data into separate images for use. However, the data for the tri-axes are horizontally stacked into one image for representation considering computational cost.

#### 3.3.2. Modified Recurrence Plot (RP)

A recurrence plot (RP) is a graphical method that shows temporal relationships in time-series data. It refers to the situation where the trajectory approaches a position or state that it has visited before. Such recurrent behavior is not easily observable in the time domain. Therefore, it can be effectively represented using the recurrence plot equation proposed by Eckmann et al. [49].

(3) $$R_{i,j}=\Theta (\epsilon -∥x_{i}-x_{j}∥)$$

Here, *x* represents the value of the time series at a given time, and Θ (·) is the Heaviside step function. Traditionally, a recurrence plot uses a Heaviside step function to calculate the distance between two vectors, which is then binarized, with a value of 0 if the distance exceeds a threshold, and 1 if it does not. However, in this study, to examine the changes and distribution of the relative temporal relationships in human activity, the binary process is omitted. Instead, the distance between each vector *x_i_* and *x_j_* is calculated and used directly as matrix *R*. This method allows for a richer relationship representation and minimized information loss in human activity, rather than just dichotomizing the relationship between the values.

Recurrence can be a key factor in understanding how signals exhibit predictable patterns or movements. By visualizing recurrence data in the form of a plot, hidden patterns, dependencies, and regularities within the data can be uncovered [22]. This is particularly useful for repetitive activities such as walking, climbing stairs, and running, where recurrent patterns can reveal underlying regularities.

While traditional RPs are typically based on a single time-series signal, accelerometer data consist of three axes. To address this, we employed a modified RP approach [21,22]. For each axis of the accelerometer data, we computed an individual recurrence plot and then combined the plots by merging the x, y, and z axes into a single RGB image. In this RGB image, each color channel corresponds to the recurrence plot of one accelerometer axis.

#### 3.3.3. Modified Multi-Channel Plot (MP)

**Figure 3:**
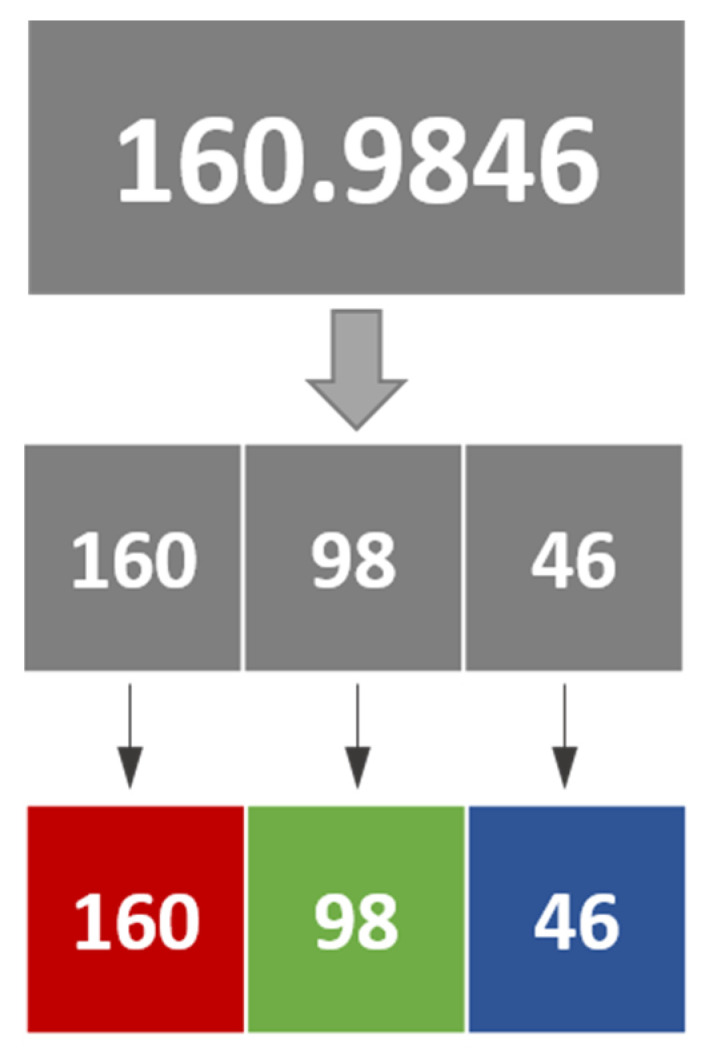
Example of process for reconstructing modified MP.

In the modified MP method, the raw accelerometer data values were normalized using a min–max scaler to scale them within the range of 0 to 255 before being mapped to an RGB image. The data values from the x, y, and z axes were then segmented into different parts: the integer, the first to second decimal places, and the third to fourth decimal places, respectively. These segmented values were assigned to the R, G, and B channels of the resulting image. To facilitate an understanding of this process, refer to Figure 3. By integrating the information from all three axes into a single image, subtle differences between activities can be visually represented. This method follows the approach presented in Hur T. et al. [50]. This process allows the continuous correlation of the signal to be reflected in the RGB channels by applying a slight distortion to the accelerometer data’s real numbers.

### 3.4. Multi-Features Fusion CNN Model

**Figure 4:**
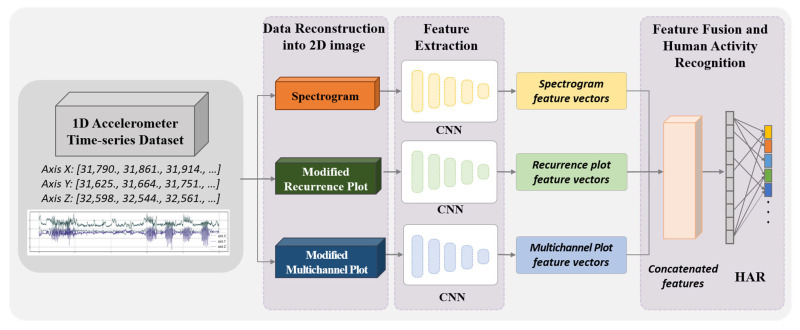
Overall architecture of proposed model.

The proposed method follows a CNN architecture designed to efficiently extract spatial features from the transformed accelerometer dataset using multiple data reconstruction methods: spectrogram, modified recurrence plot, and modified multichannel plot. Due to the nature of the transformed input data, we leverage CNNs, which possess an inductive bias that makes them highly effective for processing image-based data. The overall architecture of the model is shown in Figure 4. Each multi-input image fed into three separate CNN branches consists of 5 convolutional blocks and a fully connected layer. Each CNN processes its corresponding 2D image representation independently, generating three distinct feature vectors. These three feature vectors are concatenated into a unified representation, and the concatenated feature vector is then passed through 2 fully connected layers (units: 256, number of classes) for human activity classification. The softmax function is applied to vectors coming out from the model before the loss computation.

**Figure 5:**
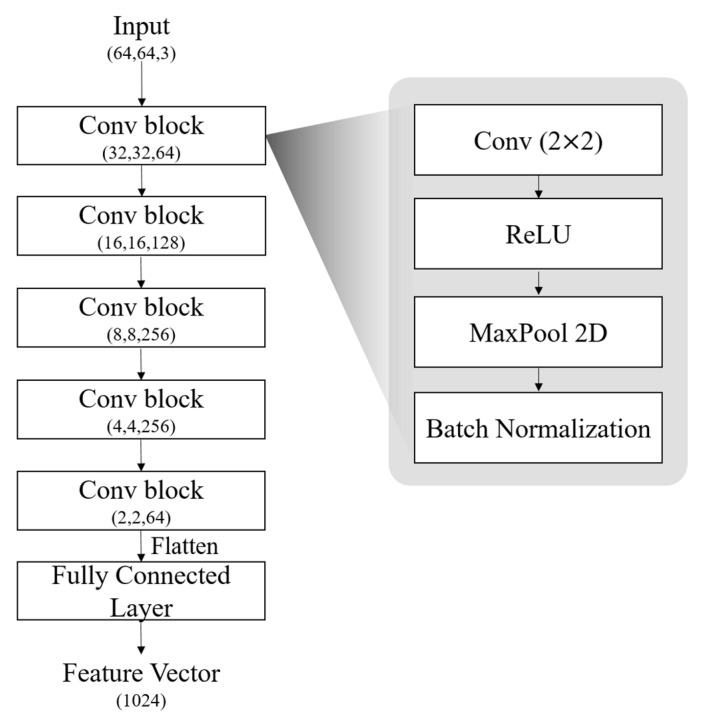
Detailed structure of convolutional blocks and CNN model.

The detailed structures of convolutional blocks and each CNN model are shown in Figure 5. The input to the network is a three-channel image size of 64 × 64 × 3. The feature extraction process consists of a series of convolutional blocks, each performing downsampling while increasing the number of feature channels. At the final convolutional block, the output feature map has a spatial size of 2 × 2 with 64 channels, resulting in a flattened feature vector size of 256. This feature vector is then passed through a fully connected layer with 1024 units to enhance the representation capacity before being fed into the feature fusion stage. The feature vectors can reflect the distinct characteristics of the reconstructed input, and these are concatenated for feature fusion. The choice of maintaining a (2, 2, 64) shape at the last convolutional layer, rather than further increasing the number of channels, is intentional to balance feature extraction efficiency and computational cost. By keeping the final feature map compact, we reduce the number of trainable parameters while preserving essential feature information. This parameter reduction is particularly crucial in HAR, where models often need to be deployed on resource-constrained devices. A smaller number of parameters leads to lower memory requirements and faster inference times, making the model more suitable for real-time HAR applications.

In HAR, lightweight and fast classification is an important factor, making it essential to select the most efficient model. Therefore, this study utilized a 5-layer simple CNN with a minimal number of parameters. To confirm that this 5-layer simple CNN is advantageous not only in terms of parameter efficiency but also in performance, its effectiveness was compared against backbones such as VGG16 [51], ResNet50 [52], MobileNet_v2 [53], and EfficientNet_b0 [54], and CNN-LSTM and CNN-GRU hybrid models.

### 3.5. Evaluation

For evaluating our multi-features fusion network, the top 1 accuracy, top 3 accuracy, precision, recall, and ROC AUC are calculated, as these parameters are widely accepted evaluations for classification tasks. Accuracy, precision, and recall are defined as Equations (4)–(6), respectively:

(4) $$Accuracy=\frac{TP+TN}{TP+FP+TN+FN}$$

(5) $$Precision=\frac{TP}{TP+FP}$$

(6) $$Recall=\frac{TP}{TP+FN}$$

where *TP* is True Positive, *TN* is True Negative, *FP* is False Positive, and *FN* is False Negative. ROC AUC evaluates the model’s ability to distinguish between classes by summarizing the trade-off between the *TP* rate and the *FP* rate across different thresholds. A higher AUC indicates better performance.

## 4. Experimental Setup

The experimental setup involved using TensorFlow 2.4.0 and Python 3.6.13 on a system equipped with Intel^®^ Core™ i7-10700K CPU and NVIDIA RTX 3090 GPU. The models were trained without data augmentation. The training was initialized with the number of epochs at 50. Additionally, the ReduceLROnPlateau function was employed to establish a mechanism where the learning rate diminishes by a factor of 0.7 if the validation loss remains stagnant for over three consecutive epochs. The size of input images is fixed at 64 × 64, and the batch size was set to eight. Categorical cross-entropy was used for the loss function. An Adam optimizer with a learning rate of 0.005 was utilized. All experiments were conducted four times, and the mean and standard deviation of the results were recorded.

## 5. Results and Discussion

### 5.1. Comparison Study on Spectrogram Window Sizes

To investigate the impact of spectrogram window size, HAR performance was compared across different window sizes. The window sizes were set to 1, 2, 5, and 10 s, with an overlap of half the window size for each setting. The experimental results (Table 3) showed that using a 10 s window size achieved the highest performance across all evaluation metrics. Particularly, the 10 s window size achieved a top one accuracy of 77.89%, representing an improvement of over 10% compared to the 1 s window. The superior performance demonstrated by the 10 s window spectrogram can be explained by several factors. Longer segments provide a greater number of data points, enabling the model to better capture overall patterns and the distinguishing characteristics of various activities. Spectrograms, which analyze signals in the time–frequency domain, benefit from longer segments, as they provide a richer representation of frequency variation patterns. Also, shorter segments are more prone to noise, which can obscure meaningful signals and degrade classification accuracy.

### 5.2. Comparison of Image Transformation Techniques

As shown in Table 4, it can be observed that the proposed method outperforms each data reconstruction method individually. To evaluate the performance of one-dimensional time-series data, 2D convolutions were replaced with 1D convolutions, and raw accelerometer data were used as a single input. Particularly, the proposed method exhibited 16.77%, 16.13%, and 19.72% improvement over the raw time-series model in terms of accuracy, precision, and recall. When comparing the performance of models based on different data reconstruction methods, the model that fused features from the three input values demonstrated the best performance. This suggests that combining features derived from each reconstructed graphic effectively captures the latent patterns and variations inherent in accelerometer data.

**Figure 6:**
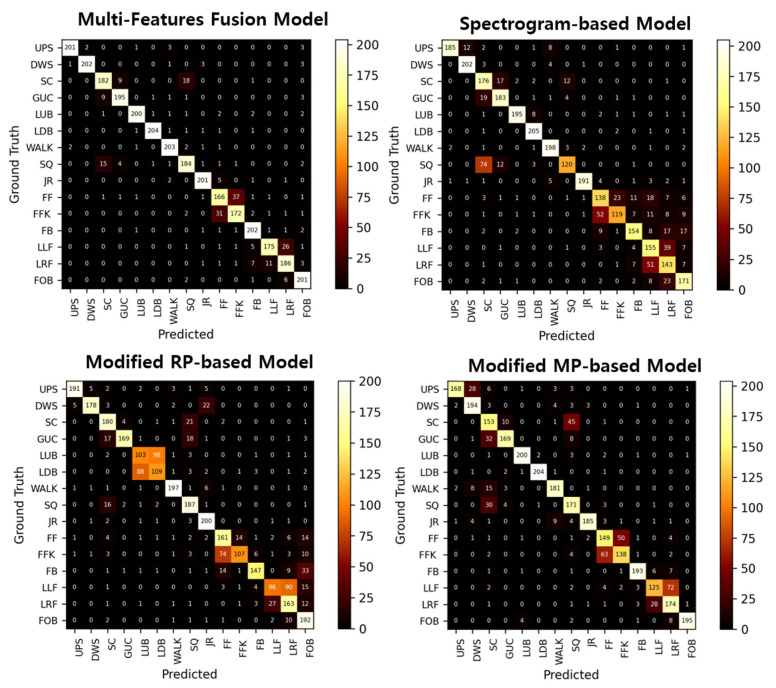
Illustration of confusion matrices.

Based on the confusion matrix shown in Figure 6, it is evident that across all models, including the proposed model, the most confusing classes areas follows: fall forward and fall forward on one’s knees. In the case of ‘fall forward’ and ‘fall forward on one’s knees’, both actions share falling forward motion elements. Therefore, the movements can be very close in terms of accelerometer data.

The spectrogram-based model exhibited notable confusion between ‘lying up on the bed’ and ‘lying down on the bed’, ‘squat and rise’, and ‘sitting on the chair’, as well as among the fall-related classes. This can be attributed to the model’s limited utilization of temporal information, which hindered its ability to effectively capture the subtle differences in brief and rapid movements. The misclassification of ‘squat and rise’ as ‘sitting on the chair’ was the most prominent issue, likely due to the similarity in their low-frequency patterns. The ‘squat and rise’ movement involves gradual changes in low-frequency components as the body lowers and rises, while the ‘sitting on the chair’ movement also generates strong low-frequency energy during the transition from standing to sitting. Furthermore, the use of a 10 s window size for STFT may have blurred finer movement details. The modified RP-based model focuses on capturing the recurrence and repetition of time-series data. The RP-based method performed poorly in distinguishing activities such as ‘lying up on the bed’ and ‘lying down on the bed’, ‘look left and fall’, and ‘look right and fall’, where the magnitudes of accelerometer data are similar, but the directional patterns are opposite. This limitation arises because the model interprets these activities as recurring patterns without effectively distinguishing their directional differences. Meanwhile, the modified MP-based model showed reduced performance in fall-related activities, likely due to the random noise and abrupt signal changes inherent to such activities. These challenges suggest that while individual models have strengths, their ability to distinguish similar activity patterns remains constrained by their respective feature representations.

In contrast, the multi-features fusion model achieved superior accuracy by integrating temporal, spatial, and frequency features, enabling it to capture nuanced movement patterns and effectively differentiate overlapping activity classes, as demonstrated in its clear separation of confusion matrix patterns. In this experiment, HAR is achievable through the 2D CNN model without preprocessing such as noise reduction in the time-series data. One-dimensional data can be reconstructed into a spectrogram, modified RP, and modified MP, allowing the extraction of the frequency domain, temporal relation, and time domain-based features, respectively. The fusion of the extracted features has the potential to markedly enhance performances.

### 5.3. Comparison of Noise Robustness

**Figure 7:**
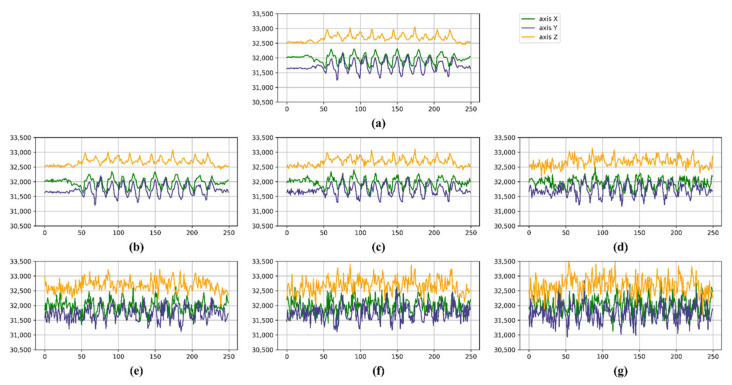
Illustration of Gaussian noise-added signal. (**a**) Original signal, (**b**) 5% noise, (**c**) 10% noise, (**d**) 20% noise, (**e**) 30% noise, (**f**) 40% noise, and (**g**) 50% noise-added signal.

To evaluate the robustness of our method against noise, we introduced Gaussian noise with different intensity levels to the test dataset. The noise levels were set to 5%, 10%, 20%, 30%, 40%, and 50% of the standard deviation (σ = 550.94) of the original data, corresponding to a σ value of [22.5, 55, 110, 165, 220, 275]. This approach allows us to systemically analyze the model’s performance across varying degrees of noise contamination. Gaussian noise was added to each value of the dataset. Figure 7 illustrates the extent of distortion introduced by the varying degrees of noise added.

**Figure 8:**
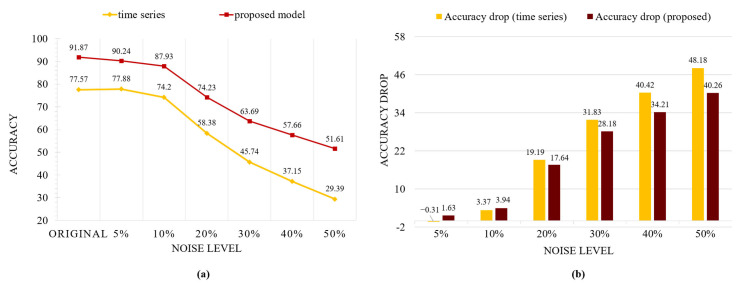
Comparison of time-series-based model and proposed models under various Gaussian noise levels. (**a**) Performance comparison; (**b**) performance degradation comparison.

The proposed model achieves higher accuracy than the time-series model in the noise-free condition and consistently maintains better performance as noise levels increase, as shown in Figure 8a. Both models degrade when more noise is introduced, and the proposed model shows a smaller drop in performance. The bar charts shown in Figure 8b quantify how much performance declines compared to the original baseline, demonstrating that the time-series model is more vulnerable to noise. Overall, these results demonstrate that our method maintains higher accuracy and stability under noisy conditions compared to conventional time-series approaches. This suggests that our approach is more robust to real-world data imperfections. These findings highlight the potential of our approach for real-world applications where data contamination is inevitable, such as clinical or daily living settings.

### 5.4. Comparison with Different Feature Extractor Backbones

To compare the performance of the backbone for the multi-input CNN, the proposed 5-layer simple CNN was evaluated alongside ImageNet pretrained VGG16, ResNet50, EfficientNet_b0, and MobileNet. Also, we tested the CNN-LSTM and CNN-GRU models. Traditional models were utilized because efficient signal processing for HAR is best achieved with smaller, faster models. As shown in Table 5, the proposed simple CNN model, which has the fewest parameters, demonstrated the highest performance.

The proposed model—built from a very simple 5-layer CNN—surpasses more complex backbones such as VGG16 and ResNet50, and even hybrid architectures like CNN-LSTM and CNN-GRU in terms of top 1 and top 3 accuracy, precision, recall, and ROC-AUC, despite using far fewer parameters. Specifically, the proposed model has around 3 million parameters, significantly lower than VGG16 (about 21.8 million), ResNet50 (about 25 million), MobileNet_v2 (about 10.5 million), EfficientNet_b0 (about 20.6 million), and CNN-LSTM/GUR (about 7 million each). Despite its streamlined design, it achieves a top 1 accuracy of 91.87%, with precision and recall both exceeding 91%.

HAR tasks often do not require highly complex models with excessive parameters [55]. Models with a large number of parameters are prone to overfitting, especially when trained on datasets that are not sufficiently large. Accelerometer data used in HAR tasks are inherently low-dimensional and represented by three axes with repetitive patterns. They lack high-frequency spatial or contextual complexity unlike natural image or text data. Complex architectures including EfficientNet or ResNet50 are designed to process such rich, high-dimensional data. However, our proposed method is designed to avoid data preprocessing steps such as filtering and normalization. The noise of the image data can inadvertently amplify noise due to their capacity to overfit fine-grained details. By keeping the architecture shallow, our model ensures that critical features remain prominent throughout the network, reducing the risk of losing important information through over-parameterization or excessive downsampling. Also, the key reason for the superior performance of the proposed model is that models like CNN-LSTM and CNN-GRU, which excel at handling sequential data, did not fully exploit their time-series advantages because the data were converted into a single image. As a result, the inherent temporal relationships that LSTM and GRU architectures had were not effectively captured.

The proposed simple CNN is specifically designed to efficiently learn the core features of the accelerometer data, resulting in a better balance between model complexity and task requirements. Thus, the superior performance of the proposed model highlights the importance of selecting an appropriate model.

## 6. Conclusions

The significance of our research lies in demonstrating that human activity recognition can be effectively achieved using a multi-features deep learning model by reconstructing time-series data without complex signal preprocessing steps. By integrating a spectrogram, modified recurrence plot, and modified multichannel plot, the proposed method successfully captures frequency, temporal, and time domain features, enhancing feature extraction and classification performance. The results highlight the superior performance of the proposed simple CNN architecture, which outperformed larger, parameter-intensive models. Through validation on our own motion dataset, we have showcased the effectiveness and robustness of this approach, offering a streamlined and efficient method for HAR that can be readily implemented in real-world applications. Additionally, this study reveals the importance of using an optimal spectrogram window size, with a 10 s window achieving the highest performance due to its ability to provide richer frequency domain information and reduce noise.

Our proposed method reduces the number of parameters, enabling faster training and inference while maintaining high performance. With an inference time of approximately 0.205 s for a 10 s data interval, the model is capable of processing data and delivering predictions promptly. This makes the model particularly suitable for real-time applications in resource-constrained environments. The lightweight design is well suited for deployment in embedded systems or mobile devices, where computational resources and energy efficiency are critical.

Further improvements in this study could lead to broader investigations. Firstly, data collection from a wider age range and the evaluation of its effectiveness in a general context. In this study, we collected data from participants aged 20 to 40 due to practical constraints. Collecting data from individuals over 40, particularly the elderly, presented several challenges. Older adults may have difficulty performing certain movements, such as squats or balance-related tasks, making data collection less feasible. Additionally, given the study timeline and available resources, we had to limit the participant age range. Future research should address this limitation by expanding the dataset to include a broader age range. Furthermore, further research is needed to develop safe methods for collecting movement data from elderly individuals without the risk of injury. Secondly, since the data collected include bioelectric signals (EMG) through the Holter ECG system, further study could explore the integration of this electric signal [33,56] to enhance the performance of HAR systems.

## Figures and Tables

**Table 1:** The demographic characteristics of subjects.

	Mean	Standard Deviation
Age (years)	27.17	4.48
Height (cm)	169.97	8.23
Weight (kg)	65.84	13.0

**Table 2:** The 15 daily activities in our dataset.

Activity	Abbreviation	Number of Samples
Going upstairs	UPS	1050
Going downstairs	DWS	1050
Sitting on a chair	SC	1050
Getting up from a chair	GC	1050
Lying up on the bed	LUB	1050
Lying down on the bed	LDB	1050
Walking	WK	1050
Squat and rise	SQ	1050
Jump rope	JR	1050
Fall forward	FF	1050
Fall forward on one’s knees	FFK	1050
Fall back	FB	1050
Look to the left and fall	LF	1050
Look to the right and fall	RF	1050
Fall out of the bed	FOB	1050

**Table 3:** Comparison on spectrogram window size.

Window Size (Seconds)	Top 1 Accuracy	Top 3 Accuracy	Precision	Recall	ROC AUC
1	67.68 (8.88)	91.98 (3.40)	69.00 (8.12)	67.68 (8.88)	96.45 (1.47)
2	70.05 (10.29)	91.20 (3.60)	71.22 (9.85)	70.05 (10.29)	96.63 (1.75)
5	74.77 (5.72)	94.58 (1.18)	76.13 (5.25)	74.77 (5.72)	97.68 (0.77)
10	77.89 (5.43)	94.89 (0.98)	78.66 (5.17)	77.86 (5.41)	98.03 (0.57)

**Table 4:** Comparison with each data reconstruction method and proposed method.

Method	Top 1 Accuracy	Top 3 Accuracy	Precision	Recall	ROC AUC
1D time series ^1^	75.23. (0.33)	91.65 (0.92)	78.44(0.14)	75.57 (0.33)	100 (0)
Spectrogram	77.89 (5.43)	94.89 (0.98)	78.66 (5.17)	77.86 (5.41)	98.03 (0.57)
Modified RP	76.32 (1.57)	95.50 (0.36)	77.22 (0.98)	76.32 (1.57)	97.71 (0.16)
Modified MP	83.82 (2.18)	96.92 (0.24)	84.43 (1.77)	83.82 (2.18)	98.90 (0.09)
Proposed method	91.87 (0.16)	97.92 (0.12)	91.97 (0.18)	91.87 (0.16)	99.85 (0.20)

^1^ Converted the 2D model architecture into a 1D model.

**Table 5:** Comparison using different feature extractor backbones.

Backbone Model	VGG16	ResNet50	MobileNet	EfficientNet	CNN-LSTM	CNN-GRU	Proposed Model
Number of parameters	21,799,759	25,034,127	10,521,167	20,571,826	7,0192,431	7,059,919	3,068,431
Top 1 accuracy	76.80 (3.95)	80.49 (1.00)	65.11 (2.76)	75.44 (2.30)	76.96 (5.25)	73.05 (4.67)	91.87 (0.16)
Top 3 accuracy	95.52 (0.02)	96.03 (0.08)	90.66 (0.00)	95.39 (0.68)	97.07 (0.44)	96.30 (1.00)	97.92 (0.12)
Precision	77.75 (3.92)	82.11 (0.58)	66.33 (1.93)	72.81 (3.02)	77.84 (5.73)	73.99 (4.87)	91.97 (0.18)
Recall	76.80 (3.95)	80.49 (1.00)	65.11 (2.76)	75.44 (2.30)	76.96 (5.25)	73.05 (4.67)	91.87 (0.16)
ROC-AUC	97.90 (0.12)	98.31 (0.00)	95.51 (0.17)	97.43 (0.14)	98.15 (0.61)	97.47 (0.70)	99.85 (0.20)

## Data Availability

The data presented in this study are available on request from the corresponding author due to privacy.
